# Tris(ethane-1,2-diamine-κ^2^ *N*,*N*′)nickel(II) 5-hy­droxy­isophthalate monohydrate

**DOI:** 10.1107/S1600536811001449

**Published:** 2011-01-26

**Authors:** Shu-Hong Wang, Bin Zhang, Cheng Wang, Guo-Qiang Xu, Yang Xie

**Affiliations:** a College of Material Science and Chemical Engineering, Harbin Engineering University, Harbin 150001, People’s Republic of China; b Key Laboratory of Polymer Functional Materials, College of Chemical Engineering and Material, Heilongjiang University, Harbin 150080, People’s Republic of China; c Institute of Petrochemistry, HLJ Academy of Sciences, Harbin 150040, People’s Republic of China

## Abstract

The asymmetric unit of the title compound, [Ni(C_2_H_8_N_2_)_3_](C_8_H_4_O_5_)·H_2_O, contains one [Ni(en)_3_]^2+^ cation (en is ethane-1,2-diamine), one 5-hy­droxy­isophthalate dianion and one water mol­ecule. In the cation, the Ni^2+^ ion is coordinated by six N atoms from three ethyl­enediamine ligands in a distorted octa­hedral geometry. The complex ions and water mol­ecules are linked by weak N—H⋯N/O and O—H⋯N/O hydrogen bonds into a three-demensional structure.

## Related literature

For the construction of supra­molecular networks, see: Colacio *et al.* (2002 ▶); Guilera & Steed (1999 ▶); Roesky & Andruh (2003 ▶). For the structures of compounds with 5-hy­droxy­isophthalic acid, see: Braverman & LaDuca (2007 ▶); Feller & Cheetham (2009 ▶); Li *et al.* (2005 ▶); Shao *et al.* (2009 ▶); Wang *et al.* (2007 ▶); Xu & Li (2004 ▶).
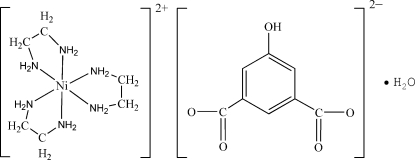


## Experimental

### 

#### Crystal data

[Ni(C_2_H_8_N_2_)_3_](C_8_H_4_O_5_)·H_2_O
*M* *_r_* = 437.13
Monoclinic, 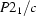

*a* = 8.208 (5) Å
*b* = 14.590 (5) Å
*c* = 16.581 (5) Å
β = 97.747 (5)°
*V* = 1967.5 (15) Å^3^
*Z* = 4
Mo *K*α radiation
μ = 1.03 mm^−1^
*T* = 293 K
0.10 × 0.08 × 0.06 mm

#### Data collection

Bruker APEX CCD area-detector diffractometer
Absorption correction: multi-scan (*SADABS*; Sheldrick, 1996 ▶) *T*_min_ = 0.906, *T*_max_ = 0.940
8306 measured reflections
3656 independent reflections
2997 reflections with *I* > 2σ(*I*)
*R*_int_ = 0.026

#### Refinement

*R*[*F* ^2^ > 2σ(*F* ^2^)] = 0.032
*wR*(*F* ^2^) = 0.092
*S* = 1.00
3656 reflections
258 parameters
4 restraints
H atoms treated by a mixture of independent and constrained refinement
Δρ_max_ = 0.45 e Å^−3^
Δρ_min_ = −0.39 e Å^−3^

### 

Data collection: *SMART* (Bruker, 2007 ▶); cell refinement: *SAINT* (Bruker, 2007 ▶); data reduction: *SAINT*; program(s) used to solve structure: *SHELXS97* (Sheldrick, 2008 ▶); program(s) used to refine structure: *SHELXL97* (Sheldrick, 2008 ▶); molecular graphics: *SHELXTL* (Sheldrick, 2008 ▶); software used to prepare material for publication: *SHELXTL*.

## Supplementary Material

Crystal structure: contains datablocks I, global. DOI: 10.1107/S1600536811001449/hy2388sup1.cif

Structure factors: contains datablocks I. DOI: 10.1107/S1600536811001449/hy2388Isup2.hkl

Additional supplementary materials:  crystallographic information; 3D view; checkCIF report

## Figures and Tables

**Table 1:** Selected bond lengths (Å)

Ni1—N5	2.112 (2)
Ni1—N4	2.120 (2)
Ni1—N2	2.123 (2)
Ni1—N3	2.129 (2)
Ni1—N6	2.135 (2)
Ni1—N1	2.139 (2)

**Table 2:** Hydrogen-bond geometry (Å, °)

*D*—H⋯*A*	*D*—H	H⋯*A*	*D*⋯*A*	*D*—H⋯*A*
O5—H4⋯O2^i^	0.82	1.80	2.618 (2)	174
N5—H14⋯O4^ii^	0.90	2.09	2.958 (3)	161
N5—H13⋯O4^iii^	0.90	2.38	3.238 (3)	159
N5—H13⋯O3^iii^	0.90	2.50	3.266 (3)	143
N4—H19⋯O6^iv^	0.90	2.30	3.173 (3)	162
N4—H20⋯O3^iii^	0.90	2.11	2.924 (3)	150
N3—H31⋯O4^ii^	0.90	2.14	3.011 (3)	162
N3—H32⋯O2^v^	0.90	2.41	3.214 (3)	148
N2—H27⋯O6^iv^	0.90	2.27	3.097 (3)	152
N2—H28⋯O1^v^	0.90	2.34	3.190 (3)	157
N6—H16⋯O5^vi^	0.90	2.42	3.292 (3)	164
N6—H15⋯O1^v^	0.90	2.49	3.285 (3)	148
O6—H5⋯O1^v^	0.82 (2)	2.28 (2)	3.076 (4)	165 (3)
O6—H6⋯O1^vi^	0.83 (2)	1.94 (2)	2.749 (3)	167 (3)
N1—H26⋯O3^iii^	0.92 (2)	2.08 (2)	2.953 (3)	158 (2)

Symmetry codes: (i) 
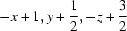
; (ii) 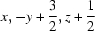; (iii) 
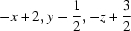
; (iv) 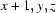; (v) 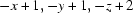; (vi) 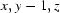.

**Fig. 1.**
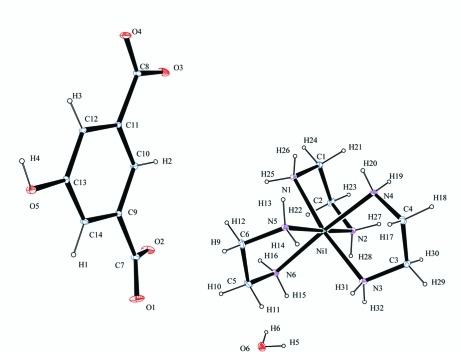
Molecular structure of [Ni(C2H8N2)3] [C8H4O5]. H2O, with the atom labeling, showing displacement at the 30% ellipsoids probability level.

**Fig. 2.**
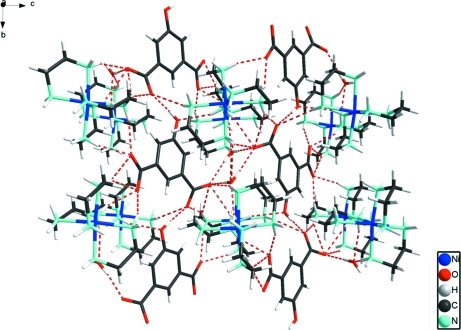
View of three-dimensional supramolecule framework of [Ni(C2H8N2)3] [C8H4O5]. H2O, with hydrogen bonds indicated by dashed lines.

## supplementary crystallographic information

### Comment

There has been considerable interest in the crystal engineering of supramolecular architectures organized and sustained by means of coordinate covalent supramolecular contacts (such as hydrogen bonds), aurophilicity interactions, and so on (Colacio *et al.*, 2002; Roesky & Andruh, 2003; Guilera & Steed, 1999). As a multidentate ligand, 5-hydroxyisophthalic acid has two rigid carboxyl groups but also one exible hydroxyl group. Therefore, 5-hydroxyisophthalic acid has been widely reported as a good candidate not only in the construction of various coordination polymers but also in the construction of supramolecular networks (Braverman & LaDuca, 2007; Feller & Cheetham, 2009; Li *et al.*, 2005; Shao *et al.*, 2009; Wang *et al.*, 2007; Xu & Li, 2004).

The molecular structure of the title compound is illustrated in Fig. 1, and selected geometric parameters are listed in Table 1. The asymmetric unit of the title compound, [Ni(C_2_H_8_N_2_)_3_] [C_8_H_4_O_5_]. H_2_O, contains one [Ni(en)_3_]^2+^ cation, one 5-hydroxyisophthalatedianion and one water molecules. In the title compound, the Ni^2+^ ion is coordinated by six N atoms from three ethylenediamine ligands in a distorted octahedral geometry. Note that a three-dimensional supramolecular hydrogen-bonding network is observed in the crystal structure of the title compound; details are given in Table 2.

### Experimental

All chemicals were purchased from commercial sources and used without further purification. A mixture of nickel nitrate (Ni(NO_3_)_2_. 3H_2_O (0.5 mmol), 5-hydroxyisophthalic acid (0.5 mmol) and ethylenediamine (0.1 mL) were dissolved in methanol (20 mL). The reaction mixture was stirred for 2 h at 313 K. The filtrate was kept at room temperature and brown block like single crystals were obtained after 3 months.

### Refinement

H atoms are treated by a mixture of independent and constrained refinement.

### Crystal data

**Table:**

[Ni(C_2_H_8_N_2_)_3_](C_8_H_4_O_5_)·H_2_O	*F*(000) = 928
*M**_r_* = 437.13	*D*_x_ = 1.476 Mg m^−^^3^*D*_m_ = 1.476 Mg m^−^^3^*D*_m_ measured by not measured
Monoclinic, *P*2_1_/*c*	Mo *K*α radiation, λ = 0.71069 Å
Hall symbol: -P 2ybc	Cell parameters from 3656 reflections
*a* = 8.208 (5) Å	θ = 2.0–51.0°
*b* = 14.590 (5) Å	µ = 1.03 mm^−^^1^
*c* = 16.581 (5) Å	*T* = 293 K
β = 97.747 (5)°	Block, brown
*V* = 1967.5 (15) Å^3^	0.10 × 0.08 × 0.06 mm
*Z* = 4	

### Data collection

**Table:**

Bruker APEX CCD area-detector diffractometer	3656 independent reflections
Radiation source: fine-focus sealed tube	2997 reflections with *I* > 2σ(*I*)
graphite	*R*_int_ = 0.026
ω scans	θ_max_ = 25.5°, θ_min_ = 1.9°
Absorption correction: multi-scan (*SADABS*; Sheldrick, 1996)	*h* = −8→9
*T*_min_ = 0.906, *T*_max_ = 0.940	*k* = −16→17
8306 measured reflections	*l* = −20→9

### Refinement

**Table:**

Refinement on *F*^2^	Primary atom site location: structure-invariant direct methods
Least-squares matrix: full	Secondary atom site location: difference Fourier map
*R*[*F*^2^ > 2σ(*F*^2^)] = 0.032	Hydrogen site location: inferred from neighbouring sites
*wR*(*F*^2^) = 0.092	H atoms treated by a mixture of independent and constrained refinement
*S* = 1.00	*w* = 1/[σ^2^(*F*_o_^2^) + (0.0592*P*)^2^ + 0.2444*P*] where *P* = (*F*_o_^2^ + 2*F*_c_^2^)/3
3656 reflections	(Δ/σ)_max_ = 0.004
258 parameters	Δρ_max_ = 0.45 e Å^−^^3^
4 restraints	Δρ_min_ = −0.39 e Å^−^^3^

### Special details

**Table:**

Geometry. All e.s.d.'s (except the e.s.d. in the dihedral angle between two l.s. planes) are estimated using the full covariance matrix. The cell e.s.d.'s are taken into account individually in the estimation of e.s.d.'s in distances, angles and torsion angles; correlations between e.s.d.'s in cell parameters are only used when they are defined by crystal symmetry. An approximate (isotropic) treatment of cell e.s.d.'s is used for estimating e.s.d.'s involving l.s. planes.
Refinement. Refinement of *F*^2^ against ALL reflections. The weighted *R*-factor *wR* and goodness of fit *S* are based on *F*^2^, conventional *R*-factors *R* are based on *F*, with *F* set to zero for negative *F*^2^. The threshold expression of *F*^2^ > σ(*F*^2^) is used only for calculating *R*-factors(gt) *etc*. and is not relevant to the choice of reflections for refinement. *R*-factors based on *F*^2^ are statistically about twice as large as those based on *F*, and *R*- factors based on ALL data will be even larger.

### Fractional atomic coordinates and isotropic or equivalent isotropic displacement parameters (Å^2^)

**Table:**

	*x*	*y*	*z*	*U*_iso_*/*U*_eq_	
Ni1	0.91637 (3)	0.259626 (17)	0.974252 (16)	0.02383 (11)	
O5	0.4795 (2)	1.20853 (10)	0.77712 (10)	0.0387 (4)	
H4	0.5319	1.2412	0.7497	0.058*	
C4	1.2188 (3)	0.3041 (2)	1.08329 (16)	0.0453 (6)	
H17	1.1993	0.3693	1.0888	0.054*	
H18	1.3343	0.2922	1.1010	0.054*	
C9	0.4722 (3)	0.96063 (13)	0.79873 (13)	0.0247 (5)	
O2	0.3689 (2)	0.81120 (11)	0.81877 (12)	0.0564 (6)	
O3	0.8221 (2)	0.88664 (12)	0.64932 (12)	0.0527 (5)	
O4	0.8811 (2)	1.03211 (12)	0.62789 (11)	0.0469 (5)	
N5	0.8745 (2)	0.40180 (13)	0.95883 (12)	0.0338 (4)	
H14	0.8949	0.4308	1.0071	0.041*	
H13	0.9411	0.4252	0.9251	0.041*	
C13	0.5152 (3)	1.11865 (13)	0.76445 (13)	0.0263 (5)	
C11	0.6694 (2)	0.99956 (13)	0.70870 (12)	0.0232 (4)	
O1	0.3348 (3)	0.91643 (12)	0.90927 (12)	0.0614 (6)	
N4	1.1751 (2)	0.27597 (14)	0.99752 (13)	0.0352 (5)	
H19	1.2254	0.2228	0.9885	0.042*	
H20	1.2080	0.3189	0.9642	0.042*	
N3	0.9414 (3)	0.26683 (14)	1.10360 (12)	0.0369 (5)	
H31	0.9101	0.3224	1.1193	0.044*	
H32	0.8776	0.2242	1.1230	0.044*	
C8	0.8013 (3)	0.97061 (15)	0.65782 (13)	0.0300 (5)	
C10	0.5897 (3)	0.93413 (14)	0.74970 (13)	0.0254 (5)	
H2	0.6146	0.8724	0.7446	0.030*	
C12	0.6296 (3)	1.09163 (13)	0.71495 (12)	0.0248 (4)	
H3	0.6800	1.1353	0.6858	0.030*	
C14	0.4373 (3)	1.05317 (14)	0.80625 (13)	0.0283 (5)	
H1	0.3609	1.0714	0.8397	0.034*	
N2	0.9402 (2)	0.11470 (13)	0.97466 (11)	0.0359 (5)	
H27	1.0404	0.0985	0.9995	0.043*	
H28	0.8642	0.0894	1.0021	0.043*	
N1	0.9271 (3)	0.24104 (13)	0.84709 (13)	0.0346 (5)	
N6	0.6543 (3)	0.25611 (13)	0.96287 (14)	0.0384 (5)	
H16	0.6125	0.2314	0.9148	0.046*	
H15	0.6214	0.2221	1.0030	0.046*	
C7	0.3851 (3)	0.89121 (15)	0.84474 (15)	0.0338 (5)	
C1	0.9958 (3)	0.14870 (17)	0.83782 (15)	0.0386 (6)	
H21	1.1139	0.1497	0.8540	0.046*	
H24	0.9747	0.1298	0.7813	0.046*	
C2	0.9176 (3)	0.08176 (16)	0.89022 (15)	0.0436 (6)	
H22	0.8012	0.0761	0.8707	0.052*	
H23	0.9679	0.0219	0.8875	0.052*	
C5	0.5970 (3)	0.35214 (19)	0.96839 (17)	0.0465 (7)	
H11	0.6052	0.3705	1.0250	0.056*	
H10	0.4828	0.3569	0.9445	0.056*	
C6	0.7013 (3)	0.41394 (18)	0.92398 (18)	0.0464 (7)	
H12	0.6869	0.3987	0.8665	0.056*	
H9	0.6686	0.4773	0.9296	0.056*	
C3	1.1144 (3)	0.25055 (18)	1.13507 (16)	0.0436 (6)	
H30	1.1393	0.1857	1.1327	0.052*	
H29	1.1369	0.2704	1.1913	0.052*	
O6	0.3136 (3)	0.07283 (15)	0.99974 (13)	0.0559 (5)	
H5	0.399 (3)	0.074 (2)	1.0317 (17)	0.067*	
H6	0.335 (4)	0.0285 (17)	0.9720 (17)	0.067*	
H26	1.001 (3)	0.2840 (15)	0.8337 (15)	0.041 (7)*	
H25	0.827 (3)	0.2482 (17)	0.8153 (16)	0.046 (8)*	

### Atomic displacement parameters (Å^2^)

**Table:**

	*U*^11^	*U*^22^	*U*^33^	*U*^12^	*U*^13^	*U*^23^
Ni1	0.02321 (18)	0.02431 (16)	0.02524 (17)	−0.00172 (10)	0.00789 (12)	0.00261 (10)
O5	0.0551 (11)	0.0185 (7)	0.0477 (10)	0.0084 (7)	0.0258 (9)	0.0027 (7)
C4	0.0298 (14)	0.0588 (17)	0.0457 (15)	−0.0068 (12)	−0.0003 (12)	−0.0092 (13)
C9	0.0259 (11)	0.0224 (10)	0.0270 (11)	−0.0007 (8)	0.0086 (9)	−0.0007 (8)
O2	0.0734 (14)	0.0239 (9)	0.0829 (14)	−0.0119 (8)	0.0509 (12)	−0.0092 (9)
O3	0.0599 (12)	0.0319 (9)	0.0750 (13)	0.0058 (8)	0.0414 (11)	−0.0119 (9)
O4	0.0528 (11)	0.0409 (10)	0.0549 (11)	0.0008 (8)	0.0363 (9)	0.0066 (8)
N5	0.0342 (11)	0.0324 (10)	0.0368 (11)	−0.0025 (8)	0.0123 (9)	0.0024 (8)
C13	0.0324 (12)	0.0182 (10)	0.0293 (11)	0.0053 (8)	0.0079 (9)	0.0007 (8)
C11	0.0242 (11)	0.0236 (10)	0.0226 (10)	0.0003 (8)	0.0058 (9)	−0.0014 (8)
O1	0.1002 (16)	0.0370 (10)	0.0593 (13)	−0.0193 (10)	0.0558 (12)	−0.0076 (8)
N4	0.0284 (11)	0.0381 (11)	0.0407 (12)	−0.0017 (8)	0.0106 (9)	0.0031 (9)
N3	0.0388 (12)	0.0384 (11)	0.0358 (11)	−0.0020 (9)	0.0136 (10)	0.0022 (8)
C8	0.0323 (12)	0.0306 (12)	0.0291 (12)	0.0021 (9)	0.0116 (10)	−0.0044 (9)
C10	0.0318 (12)	0.0174 (10)	0.0280 (11)	0.0004 (8)	0.0075 (9)	−0.0021 (8)
C12	0.0301 (11)	0.0200 (10)	0.0258 (11)	−0.0008 (8)	0.0092 (9)	0.0030 (8)
C14	0.0308 (12)	0.0258 (11)	0.0314 (12)	0.0034 (9)	0.0154 (10)	−0.0008 (9)
N2	0.0401 (12)	0.0328 (10)	0.0352 (11)	−0.0043 (9)	0.0064 (9)	0.0054 (8)
N1	0.0383 (13)	0.0343 (11)	0.0322 (11)	−0.0027 (9)	0.0086 (10)	0.0030 (8)
N6	0.0320 (12)	0.0428 (12)	0.0414 (12)	−0.0073 (8)	0.0088 (10)	0.0039 (9)
C7	0.0383 (13)	0.0235 (11)	0.0427 (14)	−0.0020 (9)	0.0174 (11)	0.0015 (9)
C1	0.0428 (14)	0.0403 (13)	0.0333 (13)	0.0041 (11)	0.0077 (11)	−0.0063 (10)
C2	0.0599 (17)	0.0281 (12)	0.0408 (14)	−0.0025 (11)	−0.0001 (13)	−0.0019 (10)
C5	0.0266 (13)	0.0508 (16)	0.0636 (18)	0.0047 (11)	0.0113 (13)	0.0016 (13)
C6	0.0364 (14)	0.0396 (14)	0.0620 (18)	0.0084 (11)	0.0016 (13)	0.0115 (12)
C3	0.0438 (16)	0.0565 (17)	0.0294 (13)	0.0077 (12)	0.0016 (12)	−0.0015 (11)
O6	0.0534 (13)	0.0568 (13)	0.0561 (14)	0.0171 (10)	0.0023 (10)	−0.0102 (10)

### Geometric parameters (Å, °)

**Table:**

Ni1—N5	2.112 (2)	N3—C3	1.464 (4)
Ni1—N4	2.120 (2)	N3—H31	0.9000
Ni1—N2	2.123 (2)	N3—H32	0.9000
Ni1—N3	2.129 (2)	C10—H2	0.9300
Ni1—N6	2.135 (2)	C12—H3	0.9300
Ni1—N1	2.139 (2)	C14—H1	0.9300
O5—C13	1.366 (2)	N2—C2	1.468 (3)
O5—H4	0.8200	N2—H27	0.9000
C4—N4	1.477 (3)	N2—H28	0.9000
C4—C3	1.510 (4)	N1—C1	1.476 (3)
C4—H17	0.9700	N1—H26	0.922 (17)
C4—H18	0.9700	N1—H25	0.922 (18)
C9—C14	1.389 (3)	N6—C5	1.485 (3)
C9—C10	1.397 (3)	N6—H16	0.9000
C9—C7	1.505 (3)	N6—H15	0.9000
O2—C7	1.245 (3)	C1—C2	1.507 (3)
O3—C8	1.248 (3)	C1—H21	0.9700
O4—C8	1.252 (3)	C1—H24	0.9700
N5—C6	1.472 (3)	C2—H22	0.9700
N5—H14	0.9000	C2—H23	0.9700
N5—H13	0.9000	C5—C6	1.502 (4)
C13—C12	1.385 (3)	C5—H11	0.9700
C13—C14	1.386 (3)	C5—H10	0.9700
C11—C10	1.386 (3)	C6—H12	0.9700
C11—C12	1.390 (3)	C6—H9	0.9700
C11—C8	1.520 (3)	C3—H30	0.9700
O1—C7	1.253 (3)	C3—H29	0.9700
N4—H19	0.9000	O6—H5	0.818 (18)
N4—H20	0.9000	O6—H6	0.826 (17)
			
N5—Ni1—N4	93.10 (8)	C11—C12—H3	119.9
N5—Ni1—N2	172.57 (8)	C13—C14—C9	120.70 (18)
N4—Ni1—N2	91.25 (8)	C13—C14—H1	119.6
N5—Ni1—N3	93.76 (8)	C9—C14—H1	119.6
N4—Ni1—N3	81.53 (8)	C2—N2—Ni1	108.85 (14)
N2—Ni1—N3	92.84 (7)	C2—N2—H27	109.9
N5—Ni1—N6	82.39 (7)	Ni1—N2—H27	109.9
N4—Ni1—N6	172.57 (8)	C2—N2—H28	109.9
N2—Ni1—N6	93.89 (8)	Ni1—N2—H28	109.9
N3—Ni1—N6	92.85 (8)	H27—N2—H28	108.3
N5—Ni1—N1	91.88 (8)	C1—N1—Ni1	106.58 (15)
N4—Ni1—N1	91.15 (8)	C1—N1—H26	108.8 (17)
N2—Ni1—N1	82.00 (7)	Ni1—N1—H26	105.5 (16)
N3—Ni1—N1	170.99 (8)	C1—N1—H25	111.6 (16)
N6—Ni1—N1	94.87 (9)	Ni1—N1—H25	113.4 (19)
C13—O5—H4	109.5	H26—N1—H25	111 (2)
N4—C4—C3	108.7 (2)	C5—N6—Ni1	107.19 (14)
N4—C4—H17	109.9	C5—N6—H16	110.3
C3—C4—H17	109.9	Ni1—N6—H16	110.3
N4—C4—H18	109.9	C5—N6—H15	110.3
C3—C4—H18	109.9	Ni1—N6—H15	110.3
H17—C4—H18	108.3	H16—N6—H15	108.5
C14—C9—C10	119.18 (18)	O2—C7—O1	122.6 (2)
C14—C9—C7	119.40 (18)	O2—C7—C9	119.42 (19)
C10—C9—C7	121.40 (18)	O1—C7—C9	118.0 (2)
C6—N5—Ni1	107.36 (14)	N1—C1—C2	109.4 (2)
C6—N5—H14	110.2	N1—C1—H21	109.8
Ni1—N5—H14	110.2	C2—C1—H21	109.8
C6—N5—H13	110.2	N1—C1—H24	109.8
Ni1—N5—H13	110.2	C2—C1—H24	109.8
H14—N5—H13	108.5	H21—C1—H24	108.2
O5—C13—C12	122.75 (18)	N2—C2—C1	109.18 (19)
O5—C13—C14	117.49 (18)	N2—C2—H22	109.8
C12—C13—C14	119.70 (18)	C1—C2—H22	109.8
C10—C11—C12	119.87 (18)	N2—C2—H23	109.8
C10—C11—C8	119.97 (18)	C1—C2—H23	109.8
C12—C11—C8	120.15 (18)	H22—C2—H23	108.3
C4—N4—Ni1	108.26 (14)	N6—C5—C6	109.3 (2)
C4—N4—H19	110.0	N6—C5—H11	109.8
Ni1—N4—H19	110.0	C6—C5—H11	109.8
C4—N4—H20	110.0	N6—C5—H10	109.8
Ni1—N4—H20	110.0	C6—C5—H10	109.8
H19—N4—H20	108.4	H11—C5—H10	108.3
C3—N3—Ni1	107.96 (15)	N5—C6—C5	108.6 (2)
C3—N3—H31	110.1	N5—C6—H12	110.0
Ni1—N3—H31	110.1	C5—C6—H12	110.0
C3—N3—H32	110.1	N5—C6—H9	110.0
Ni1—N3—H32	110.1	C5—C6—H9	110.0
H31—N3—H32	108.4	H12—C6—H9	108.3
O3—C8—O4	124.9 (2)	N3—C3—C4	108.1 (2)
O3—C8—C11	117.01 (19)	N3—C3—H30	110.1
O4—C8—C11	118.07 (19)	C4—C3—H30	110.1
C11—C10—C9	120.22 (18)	N3—C3—H29	110.1
C11—C10—H2	119.9	C4—C3—H29	110.1
C9—C10—H2	119.9	H30—C3—H29	108.4
C13—C12—C11	120.28 (18)	H5—O6—H6	99 (3)
C13—C12—H3	119.9		
			
N4—Ni1—N5—C6	168.22 (16)	C10—C9—C14—C13	1.4 (3)
N2—Ni1—N5—C6	42.5 (6)	C7—C9—C14—C13	−179.8 (2)
N3—Ni1—N5—C6	−110.08 (16)	N5—Ni1—N2—C2	23.7 (6)
N6—Ni1—N5—C6	−17.70 (16)	N4—Ni1—N2—C2	−102.10 (16)
N1—Ni1—N5—C6	76.96 (16)	N3—Ni1—N2—C2	176.32 (16)
C3—C4—N4—Ni1	40.3 (2)	N6—Ni1—N2—C2	83.27 (16)
N5—Ni1—N4—C4	80.33 (17)	N1—Ni1—N2—C2	−11.11 (16)
N2—Ni1—N4—C4	−105.70 (17)	N5—Ni1—N1—C1	166.69 (16)
N3—Ni1—N4—C4	−13.01 (16)	N4—Ni1—N1—C1	73.56 (16)
N6—Ni1—N4—C4	28.1 (7)	N2—Ni1—N1—C1	−17.54 (16)
N1—Ni1—N4—C4	172.28 (17)	N3—Ni1—N1—C1	38.0 (6)
N5—Ni1—N3—C3	−109.45 (16)	N6—Ni1—N1—C1	−110.79 (16)
N4—Ni1—N3—C3	−16.88 (15)	N5—Ni1—N6—C5	−11.55 (16)
N2—Ni1—N3—C3	73.96 (16)	N4—Ni1—N6—C5	41.3 (7)
N6—Ni1—N3—C3	168.00 (16)	N2—Ni1—N6—C5	174.91 (16)
N1—Ni1—N3—C3	19.1 (6)	N3—Ni1—N6—C5	81.87 (17)
C10—C11—C8—O3	6.3 (3)	N1—Ni1—N6—C5	−102.80 (17)
C12—C11—C8—O3	−174.4 (2)	C14—C9—C7—O2	155.0 (2)
C10—C11—C8—O4	−173.5 (2)	C10—C9—C7—O2	−26.2 (4)
C12—C11—C8—O4	5.8 (3)	C14—C9—C7—O1	−26.6 (4)
C12—C11—C10—C9	−1.4 (3)	C10—C9—C7—O1	152.2 (2)
C8—C11—C10—C9	177.87 (19)	Ni1—N1—C1—C2	43.2 (2)
C14—C9—C10—C11	−0.5 (3)	Ni1—N2—C2—C1	37.7 (2)
C7—C9—C10—C11	−179.3 (2)	N1—C1—C2—N2	−55.4 (3)
O5—C13—C12—C11	175.5 (2)	Ni1—N6—C5—C6	38.8 (2)
C14—C13—C12—C11	−1.6 (3)	Ni1—N5—C6—C5	44.1 (2)
C10—C11—C12—C13	2.5 (3)	N6—C5—C6—N5	−56.6 (3)
C8—C11—C12—C13	−176.8 (2)	Ni1—N3—C3—C4	43.4 (2)
O5—C13—C14—C9	−177.6 (2)	N4—C4—C3—N3	−56.7 (3)
C12—C13—C14—C9	−0.3 (4)		

### Hydrogen-bond geometry (Å, °)

**Table:**

*D*—H···*A*	*D*—H	H···*A*	*D*···*A*	*D*—H···*A*
O5—H4···O2^i^	0.82	1.80	2.618 (2)	174
N5—H14···O4^ii^	0.90	2.09	2.958 (3)	161
N5—H13···O4^iii^	0.90	2.38	3.238 (3)	159
N5—H13···O3^iii^	0.90	2.50	3.266 (3)	143
N4—H19···O6^iv^	0.90	2.30	3.173 (3)	162
N4—H20···O3^iii^	0.90	2.11	2.924 (3)	150
N3—H31···O4^ii^	0.90	2.14	3.011 (3)	162
N3—H32···O2^v^	0.90	2.41	3.214 (3)	148
N2—H27···O6^iv^	0.90	2.27	3.097 (3)	152
N2—H28···O1^v^	0.90	2.34	3.190 (3)	157
N6—H16···O5^vi^	0.90	2.42	3.292 (3)	164
N6—H15···O1^v^	0.90	2.49	3.285 (3)	148
O6—H5···O1^v^	0.82 (2)	2.28 (2)	3.076 (4)	165 (3)
O6—H6···O1^vi^	0.83 (2)	1.94 (2)	2.749 (3)	167 (3)
N1—H26···O3^iii^	0.92 (2)	2.08 (2)	2.953 (3)	158 (2)

Symmetry codes: (i) −*x*+1, *y*+1/2, −*z*+3/2; (ii) *x*, −*y*+3/2, *z*+1/2; (iii) −*x*+2, *y*−1/2, −*z*+3/2; (iv) *x*+1, *y*, *z*; (v) −*x*+1, −*y*+1, −*z*+2; (vi) *x*, *y*−1, *z*.
